# Recalibrating the kidney failure risk equation for a Mediterranean European population: reducing age and sex inequality

**DOI:** 10.3389/fmed.2024.1497780

**Published:** 2025-01-29

**Authors:** Daniel Bundó-Luque, Oriol Cunillera-Puértolas, Sílvia Cobo-Guerrero, José Romano, Ariadna Arbiol-Roca, José Alberto Domínguez-Alonso, Josep Maria Cruzado, Betlem Salvador-González

**Affiliations:** ^1^CAP Alt Penedès, Gerència d’Atenció Primària i a la Comunitat del Penedès, Institut Català de la Salut, Vilafranca del Penedès, Barcelona, Spain; ^2^Disease, Cardiovascular Risk, and Lifestyle in Primary Health Care (MARCEVAP) Research Group, Fundació Institut Universitari per a la recerca a l’Atenció Primària (IDIAPJGol), L’Hospitalet Llobregat, Barcelona, Spain; ^3^Facultat de Medicina, Universitat de Barcelona (UB), Barcelona, Spain; ^4^Unitat de Suport a la Recerca Metropolitana Sud, Fundació Institut Universitari per a la recerca a l’Atenció Primària de Salut Jordi Gol i Gurina (IDIAPJGol), l’Hospitalet de Llobregat, Barcelona, Spain; ^5^EAP Gavarra, Gerència d’Atenció Primària i a la Comunitat Llobregat, Institut Català de la Salut, Cornellà de Llobregat, Barcelona, Spain; ^6^EAP Sant Josep, Gerència d’Atenció Primària i a la Comunitat Delta Llobregat, Institut Català de la Salut, l’Hospitalet de Llobregat, Barcelona, Spain; ^7^Laboratori Clínic Territorial Metropolitana Sud, Hospital Universitari de Bellvitge, Institut Català de la Salut, l’Hospitalet de Llobregat, Barcelona, Spain; ^8^CAP Vilafranca Nord, Gerència d’Atenció Primària i a la Comunitat del Penedès, Institut Català de la Salut, Vilafranca del Penedès, Barcelona, Spain; ^9^Department of Nephrology, Hospital Universitari Bellvitge, l’Hospitalet de Llobregat, Barcelona, Spain; ^10^Nephrology and Renal Transplantation Group, Bellvitge Institute for Biomedical Research (IDIBELL), l’Hospitalet de Llobregat, Barcelona, Spain

**Keywords:** kidney failure, KFRE, risk assesement, age, sex, competing risk

## Abstract

**Introduction:**

Chronic kidney disease (CKD) patients may develop kidney failure (KF), receiving renal replacement therapy (RRT) in some cases. The Kidney Failure Risk Equation (KFRE-4), predicting RRT risk, is widely validated but not in a primary care Mediterranean European population. We aim to recalibrate KFRE-4 accordingly, considering death as a competing risk, to improve performance. Additionally, we recalibrate KFRE-4 for predicting KF, including all patients reaching CKD stage 5, not just those on RRT.

**Methods:**

Retrospective cohort study including individuals aged ≥50 years with confirmed glomerular filtration rate (eGFR) <60 mL/min/1.73m^2^ and measured albumin-to-creatinine ratio (ACR). Dataset was split into training and test sets. New KFRE-4 models were developed in the training set and performance was evaluated in the test set: Base hazard adapted-KFRE (Basic-RRT), Cox reestimation (Cox- RRT), Fine and Gray RRT reestimation (FG-RRT), and Fine and Gray KF reestimation (FG-KF).

**Results:**

Among 165,371 primary care patients (58.1% female; mean age 78.1 years; mean eGFR 47.3 mL/min/1.73m^2^, median ACR 10.1 mg/g), original KFRE-4 showed good discrimination but poor calibration, overestimating RRT risk. Basic-RRT showed poorer performance. Cox-RRT and FG-RRT, enhancing the influence of old age and female sex, diminished overprediction. FG-RRT, considering death as a competing risk, resulted the best RRT model. Age and sex had less impact on KF prediction.

**Conclusion:**

A fully tailored recalibration model diminished RRT overprediction. Considering death as a competing event optimizes performance. Recalibrating for KF prediction offers a more inclusive approach in primary care, addressing the needs of women and elderly.

## 1 Introduction

Chronic kidney disease (CKD) affects approximately 9.1% of the global population and is a significant contributor to mortality and morbidity (1). CKD is classified based on estimated glomerular filtration rate (eGFR) and albuminuria using the “Kidney Disease: Improving Global Outcomes” (KDIGO) stages (2). Patients with an eGFR<15 are categorized as CKD-G5 and may necessitate renal replacement therapy (RRT), such as dialysis or transplantation, which constitutes over half of the healthcare costs associated with CKD (3).

The Kidney Failure Risk Equation (KFRE), developed in 2011, was initially designed to predict the risk of RRT at 1, 3, and 5 years in a Canadian nephrology service population (4). Various predictive models were explored, with the 4-variable (KFRE-4: age, sex, albumin-to-creatinine ratio (uACR), and eGFR) and 8-variable (KFRE-8: incorporating additional variables such as calcium, phosphate, albumin, and bicarbonate in serum) models demonstrating superior performance. Subsequent multinational validations revealed the need for recalibration to adapt to local populations, with the KFRE-4 being the simplest and most widely applicable model (5).

KFRE-4 has been validated across diverse populations and specific subgroups of patients followed in nephrology services (4, 6–18). Furthermore, it has shown utility in population-based studies in the United States, Australia, and at the multinational level (5, 19–21). Its application in UK primary care settings has demonstrated promising results (22), with subsequent validations in Canada, UK, and Singapore (23–25).

In primary care, KFRE-4 assessing the 5-year risk of RRT serves as a valuable tool for guiding nephrology referrals without increasing costs (24–27). Different threshold values have been proposed (23–28). This has influenced primary care clinical guidelines such as NICE 2021 (29, 30).

However, some challenges remain to be tackled, including the overestimation of the RRT risk, particularly with advancing age and longer-term assessments, probably due to the consideration of death as censoring rather than a competing event in the estimation of prediction models (11, 16, 31). Consequently, a modified KFRE-4 equation recalibrated for competing risks, which also included eGFR splines, was proposed in 2023. It showed increased performance, especially in subgroups of older individuals or high eGFR (21). It remains uncertain if the improvement can be attributed to the competing risks or to the splines.

CKD primary care population is older and shows lower risk of CKD progression than patients followed by nephrologists (22, 24), entailing unique challenges for applying such risk prediction models. Thus, a recalibrated KFRE-4 that accounts for death as a competing risk may be more suitable for risk prediction in this population.

Validation studies conducted in population-based or primary care settings used RRT as primary outcome (5, 20–24). However, not all patients diagnosed with CKD-G5 undergo RRT (CKD-G5 without RRT); many are managed conservatively. In some studies, this treatment option accounts for up to one-third of cases (32). Thus, the primary outcome Kidney failure (KF), internationally defined as CKD-G5 or RRT (33) is more appropriate than RRT to assess the overall CKD progression risk, essential for optimizing clinical management, delaying disease progression, and minimizing costs (34). Given that both, RRT and CKD-G5 without RRT, share risk factors albeit with different weights, it is possible to validate and recalibrate KFRE for KF (32). Only one article in primary care has tried to validate KFRE for KF (25) but none has compared both outcomes in the same cohort or used the competing risk framework for validation beside development. Moreover, KFRE-4 has not been validated in a Mediterranean population yet.

This study aims to validate and recalibrate the standard KFRE-4 to assess the risk of progression to RRT in a European Mediterranean primary care population. Considering death as a competing risk during the recalibration process and validation, it is expected an improved performance. Additionally, it seeks to recalibrate a new KFRE-4 equation for predicting KF in the same cohort.

## 2 Materials and methods

This CKD primary care cohort observational study is based on secondary data. The main source is the Information System for the Development of Research in Primary Care (SIDIAP) with information about 5.8 million people (75% of the Catalan population) attended by the Catalan Health Institute (ICS), the main healthcare provider in Catalonia (North-east Spain). SIDIAP contains data from primary care electronic health registers including sociodemographic data, clinical diagnoses, laboratory results and vital status at follow-up (alive, transfer or death without specified cause). RRT data was collected from the Catalan Registry of Renal Patients (RMRC), a compulsory-notification registry on all patients undergoing RRT.

We included individuals aged ≥50 years, with confirmed eGFR <60 mL/min/1.73m^2^ (consecutive measures recorded >90 days apart) between 1st January 2010 and 31st December 2017, and at least one uACR measurement 1 year around the confirming measure. We excluded individuals with CKD-G5 (defined as confirmed eGFR <15 mL/min/1.73m^2^), dialysis or kidney transplantation at baseline, and patients within a follow-up period of <30 days.

KFRE-4 models were adapted and validated for predicting RRT -defined as dialysis or transplant, with available data until 31st December 2018 - and KF-defined as confirmed CKD-G5 (available data until 31st December 2017) or RRT. Risk at 5 years was assessed for both outcomes. Equation variables were age, sex (“1” if male or “0” if female), eGFR and uACR. Serum creatinine concentration standardized against Isotope Dilution Mass Spectrometry was utilized. The eGFR was calculated using the new Chronic Kidney Disease Epidemiology Collaboration (CKD-EPI 2021) formula without racial correction (35). Variables are concisely described in the Supplementary methods.

The dataset was divided into two random subsets (single split): one for model development (training set) and the other one for model/risk validation (test set). In the training set, we estimated 3 models explained by the variables age, sex, eGFR and uACR: (1) a Cox proportional hazards regression model to predict RRT (Cox-RRT); (2) a Fine and Gray competing risk model to predict RRT (FG-RRT); and (3) a Fine and Gray competing risk model to predict KF (FG-KF), using all available data from the inclusion in the study and censoring unobserved events at 31st December 2017. Cox models considered death, end of the study, or losses to follow up before main outcome as censoring. Fine and Gray models, on the other hand, considered death before the main outcome as a competing event, but the end of the study or losses to follow up before RRT as censoring. We also derived the base hazard at 5 years for the Tangri’s “reference individual” (aged 70.36, “a 56.42% male,” eGFR = 36.11 mL/min/1.73m^2^ and uACR = 170.2 mg/g) from the Cox-RRT model and used this for a basic KFRE-4 recalibration model (Basic-RRT). For this model we kept unchanged all parameters of the KFRE-4 non-North American equation (5) just modifying the base hazard. In the test set, we calculated the risk according to the KFRE-4 non-North American equation (5), to the Basic-RRT, to the Cox-RRT, to the FG-RRT and to the FG-KF models to assess the performance of the prediction models. In this validation of the equation, the observed risk was assessed using a competing risk approach (36).

The model’s performance was assessed by analyzing discrimination and calibration. Model discrimination describes the capacity of the model in separating individuals experiencing an event from those who do not. We used the inverse probability of censoring weighting approach to estimate the C-statistic (generally known as area under the ROC curve) for censored events with competing risks (37). The calibration of a model refers to how far the observed risks differ from the predicted ones. We used the Brier Score and calibration plots to evaluate each model, estimating the observed frequencies with the Aalen-Johansen method (36). All models were validated for predicting both RRT and KF risk at 5 years.

Analyses were performed using R-4.3.2 with riskRegression package.

This study was approved by the Ethics Committee of IDIAP Jordi Gol (P18/086- date of approval: 26 September 2018).

## 3 Results

### 3.1 Baseline population characteristics

**Figure 1:**
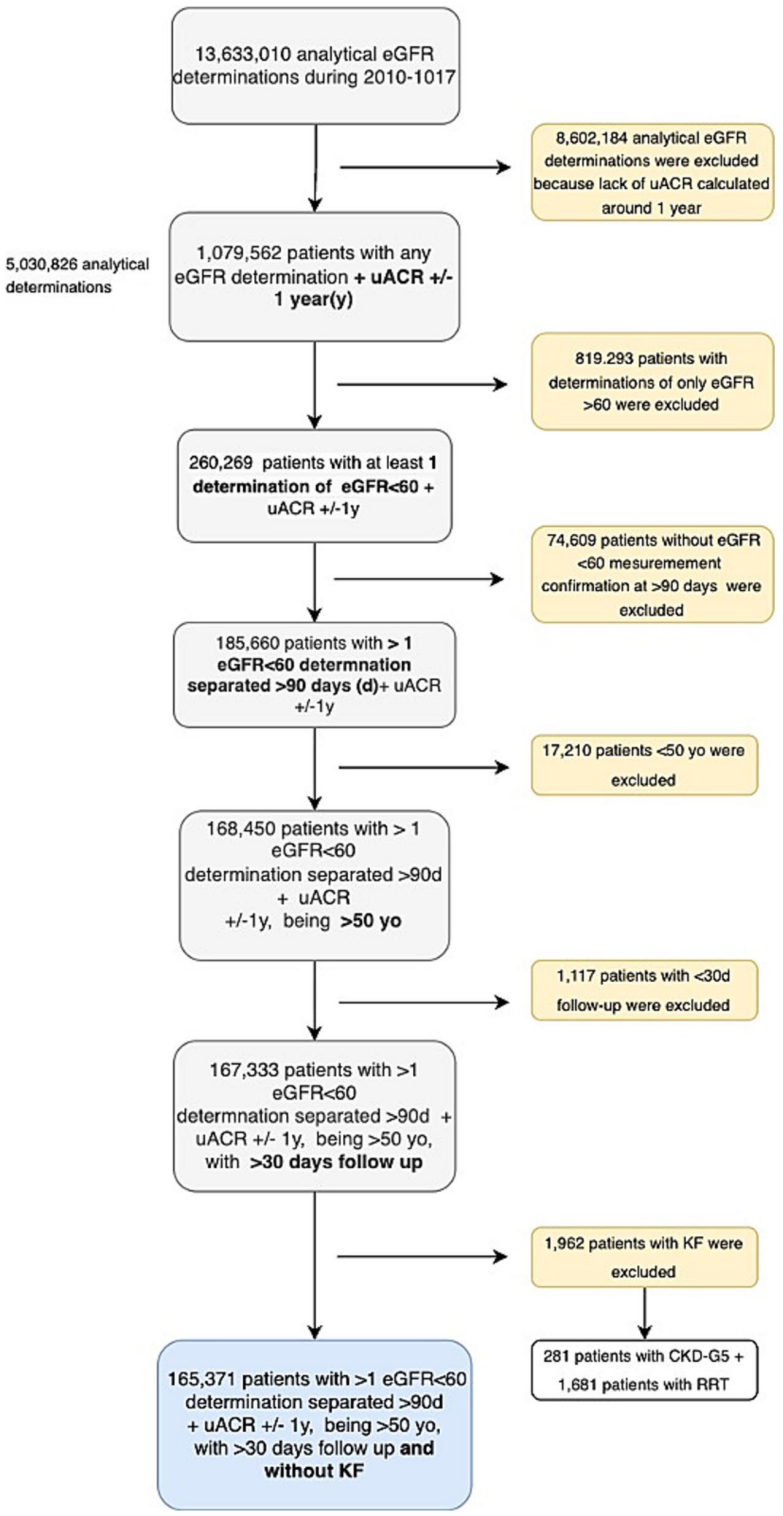
Flowchart of sample selection.

In this study, we examined a cohort comprising 165,371 patients (82,987 in the training set and 82,384 in the test set) with CKD followed in primary care centers. Patients were selected according to Figure 1.

The population was predominantly female (58.1%). The mean age was 78.1 years old, with only 9.1% of individuals in the 50–65 year range. Cardiovascular comorbidities were frequent (Table 1). Baseline characteristics of training set and development set are shown in Supplementary Table S1. Mean eGFR at baseline was 47.3 mL/min/ 1.73m^2^, with 93.3% of patients having an eGFR >30 mL/min/ 1.73m^2^. The median ACR was 10.1 mg/g, and only 5.9% of the population had values exceeding 300 mg/g (Table 2).

**Table 1:** Baseline demographics and comorbidities by KDIGO eGFR group (*n* = 165.371).

		Global (*n* = 165,371)	G3a (*n* = 109,247)	G3b (*n* = 44,914)	G4 (*n* = 10,626)	G5 (*n* = 584)	*p*-value
Age group distribution (years)	(50–65]	15,108 (9.1%)	11,004 (10.1%)	3,099 (6.9%)	909 (8.6%)	96 (16.4%)	<0.001
	(65, 75]	41,493 (25.1%)	30,901 (28.3%)	8,670 (19.3%)	1798 (16.9%)	124 (21.2%)	
	(75, 85]	74,726 (45.2%)	49,119 (45.0%)	20,898 (46.5%)	4,483 (42.2%)	226 (38.7%)	
	>85	34,044 (20.6%)	18,223 (16.7%)	12,247 (27.3%)	3,436 (32.3%)	138 (23.6%)	
Age (years)	Mean (SD)	78.1 (8.86)	77.2 (8.71)	79.9 (8.69)	80.3 (9.57)	77.0 (10.7)	<0.001
	Median [IQR]	79 [73, 84]	78 [72, 83]	81 [75, 86]	82 [75, 87]	79.5 [69, 85]	<0.001
Sex	Female	96,095 (58.1%)	62,132 (56.9%)	27,263 (60.7%)	6,403 (60.3%)	297 (50.9%)	<0.001
T1D		1,097 (0.7%)	641 (0.6%)	325 (0.7%)	124 (1.2%)	7 (1.2%)	<0.001
T2D		68,469 (41.4%)	44,914 (41.1%)	18,789 (41.8%)	4,509 (42.4%)	257 (44.0%)	0.004
Hypertension		145,067 (87.7%)	94,754 (86.7%)	40,308 (89.7%)	9,499 (89.4%)	506 (86.6%)	<0.001
CHD		27,552 (16.7%)	16,907 (15.5%)	8,342 (18.6%)	2,187 (20.6%)	116 (19.9%)	<0.001
Stroke		14,992 (9.1%)	9,086 (8.4%)	4,627(10.3%)	1,206 (11.4%)	73 (12.5%)	<0.001
PAD		12,559 (7.6%)	7,551 (6.9%)	3,900 (8.7%)	1,042 (9.8%)	66 (11.3%)	<0.001
HF		22,228 (13.4%)	11,707 (10.7%)	7,864 (17.5%)	2,531 (23.8%)	126 (21.6%)	<0.001

T1D, Type 1 Diabetes Mellitus; T2D, Type 2 Diabetes Mellitus; CHD, Coronary heart disease; PAD, Peripheral arterial disease; HF, Heart failure.

**Table 2:** Global kidney status at baseline.

		Global (*n* = 165,371)	*p*-value
Baseline eGFR (mL/min/1.73m^2^)	Mean (SD)	47.3 (9.8)	<0.001
	Median [IQR]	49.6 [41.7, 55.0]	<0.001
KDIGO eGFR groups (mL/min/1.73m^2^)	[45–60)	109,247 (66.1%)	<0.001
	[30–45)	44,914 (27.2%)	
	[15–30)	10,626 (6.43%)	
	<15	584 (0.35%)	
Baseline uACR (mg/g)	Mean (SD)	84.9 (270.4)	<0.001
	Median [IQR]	10.3 [4.0, 40.0]	<0.001
KDIGO uACR groups (mg/g)	<30	117,061 (70.8%)	<0.001
	[30, 300)	37,851 (22.9%)	
	> = 300	10,459 (6.3%)	

eGFR, estimated glomerular filtration rate; uACR: Albumin-to-Creatinine ratio.

### 3.2 Progression to KRT, CKD-G5, KF or death at follow-up

With a mean follow-up duration of 3.49 years (3rd quartile 5.1), 2,517 patients (1.5%) received RRT (1.2% at 5 years), while 593 individuals (0.4%) developed CKD-G5 without RRT during follow-up. Combining both entities, a total of 3,110 patients (1.9%) achieved the outcome KF (1.5% at 5 years). Global mortality at the end of the study was 21.8%, dying most of them before developing KF (21.2%) (Table 3). Incidence rates per 1,000 person-years were 4.39 for RRT and 5.42 for KF.

**Table 3:** Progression at follow-up and incidences.

		Global (n = 165,371)	*P*-value
Follow-up time	Mean (SD)	3.49 (2.05)	<0.001
	Median [IQR]	3.27 [1.78, 5.10]	
Evolution to RRT at follow-up			
	RRT	2,517 (1.5%)	
	Death before RRT	35,464 (21.4%)	
	Neither RRT nor death	127,390 (77.0%)	
Evolution to KF at follow-up			
	KF	3,110 (1.9%)	
	Death before KF	35,131 (21.2%)	
	Neither KF nor death	127,130 (76.9%)	
RRT at 5 years	*n* (%)	2005 (1.2%)	
KF at 5 years	*n* (%)	2,555 (1.5%)	
RRT incidence rate	1,000 person-years	4.39 (4.22, 4.56)	
KF incidence rate	1,000 person-years	5.42 (5.23, 5.62)	
Overall mortality rate	1,000 person-years	62.8 (62.1, 63.4)	

RRT, Renal replacement therapy; KF, Kidney failure; CKDG5, Stage G5 of KDIGO guidelines.

### 3.3 New KFRE models development

All adjusted models provide an estimation of the 5-year risk according to the following general formula:


$$\mathrm{Risk}=1-\mathrm{base}.\mathrm{surviva}l^{\text{exp}}\left(\begin{array}{l} coef_{\left(\mathrm{age}/10\right)}\;\times \left(\frac{\mathrm{age}}{10}-7.036\right)+coef_{\left(\mathrm{sex}=\mathrm{male}\right)}\times \\ \left(I-0.5642\right)+coef_{\left(\mathrm{eGFR}/5\right)}\times \left(\frac{\mathrm{eGFR}}{5}-7.222\right)+\\ coef_{\left(\ln\left(\mathrm{uACR}\right)\right)}\times \left(\ln\left(\mathrm{uACR}\right)-5.137\right) \end{array}\right)$$

*I = 1 if male and 0 if female.*

*Base.hazard = 1–base.survival*

The base hazard and coefficients (marginal effects) were estimated for the different models (Table 4).

**Table 4:** Parameters of the different KFRE models in the SIDIAP 2010–2017 sample.

KFRE model	Base hazard	Marginal effects			
		Age/10	Sex = Male	eGFR/5	ln (uACR)
Non-USA original KFRE	0.0635	−0.2201	0.2467	−0.5567	0.4510
Base hazard adapted-KFRE (Basic-RRT)	0.0847	−0.2201	0.2467	−0.5567	0.4510
Cox complete reestimation (Cox-RRT)	0.0847	−0.6739	0.4587	−0.5579	0.6423
Fine and Gray RRT reestimation (FG-RRT)	0.0672	−0.8301	0.3947	−0.5017	0.5662
Fine and Gray KF reestimation (FG-KF)	0.0839	−0.6618	0.2322	−0.5677	0.5441

Basic-RRT resulted in an increased base hazard of 0.0847 for RRT, instead of the 0.0635 of the non-USA population. However, when considering competing risks, this base hazard moved to 0.0672. For the new KF outcome (FG-FK) it raised to 0.0839.

The weight of the 4 explanatory variables varied heterogeneously in our sample. While there were small variations in the effect of eGFR (i.e.: −0.5017 for FG-KRT and − 0.5677 for FG-KF in comparison to the original −0.5567 effect) and uACR, the effects of age and gender were noticeably stronger in the study population. The impact of sex increased, with a marginal effect of 0.3947 for FG-RRT compared to the 0.2467 of the original non-USA equation. The marginal effect of age was estimated as −0.8301 in the FG-RRT model whereas it was −0.2201 in the original non-USA equation. This age and sex differences were mitigated in the FG-KF model.

### 3.4 Performance evaluation of different KFRE models

**Figure 2:**
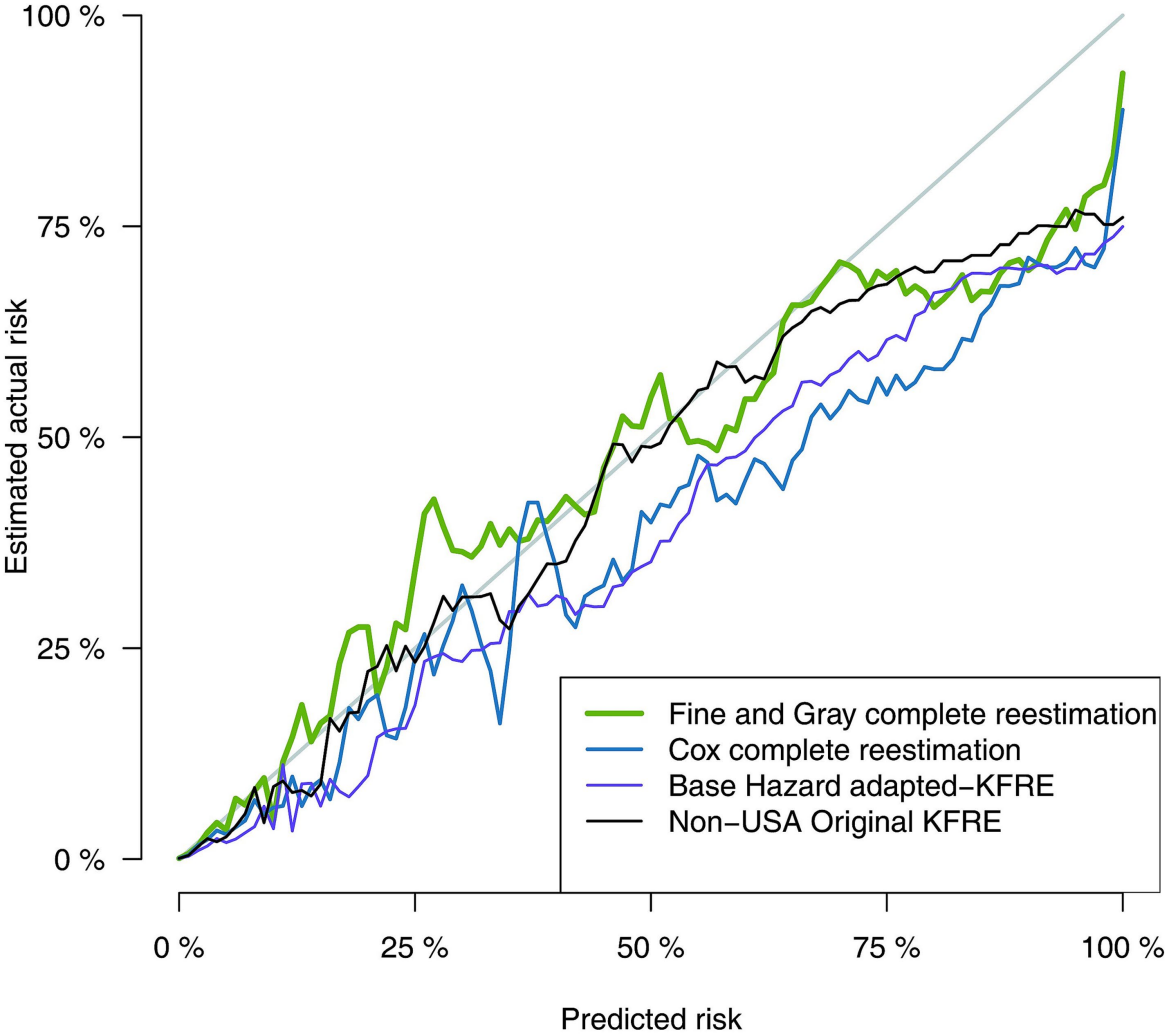
Comparison of predicted vs. observed risk using calibration plot graphical analysis.

**Figure 3:**
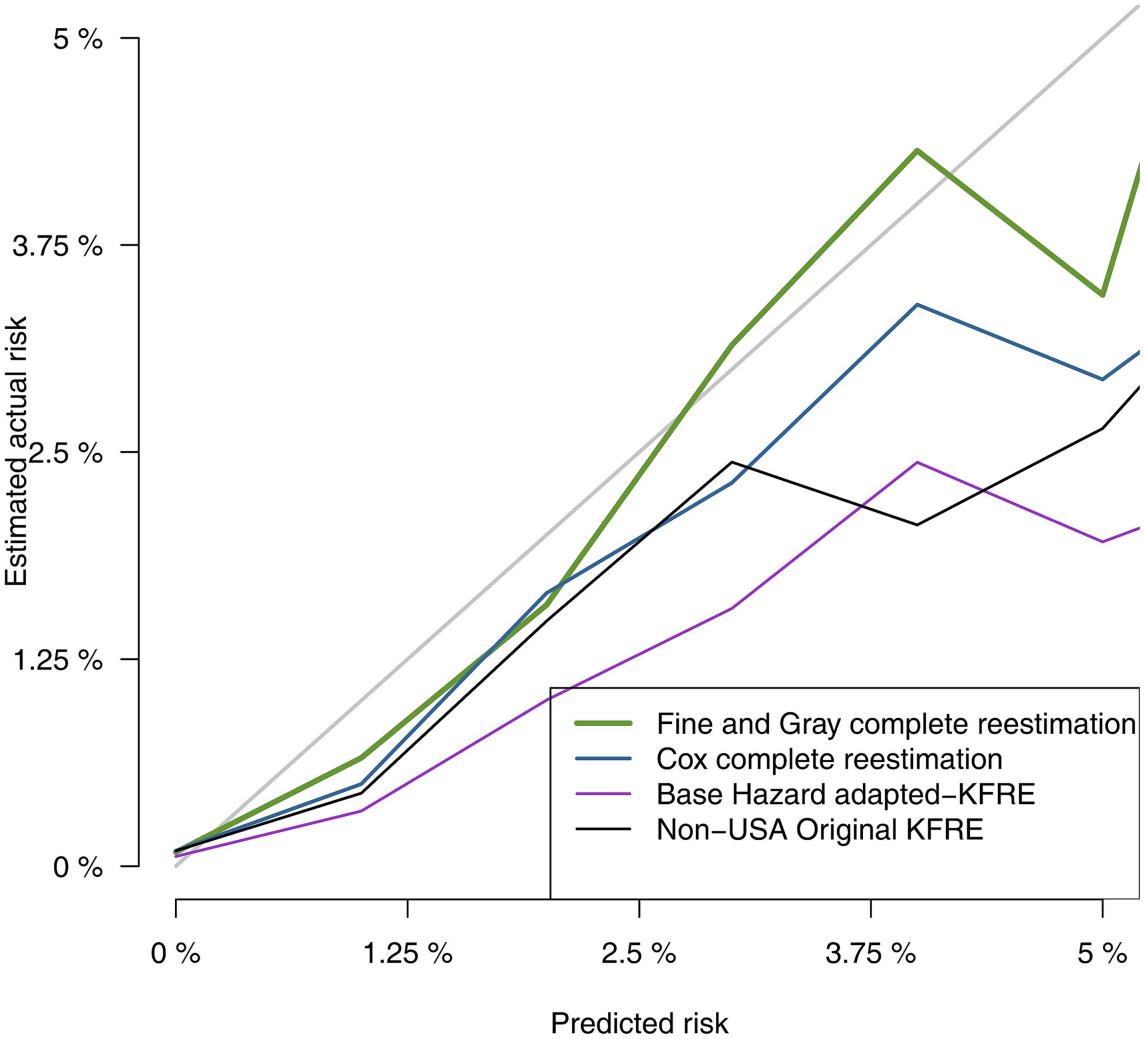
Comparison of predicted vs. observed risk in predicted low risk patients.

Discrimination and calibration statistics of the different approaches to estimate risk of RRT are presented in Table 5. All models showed good discrimination capacity, with the original KFRE-4 equation and Basic-RRT presenting the lowest C-statistic, 0.932 (95% CI 0.930, 0.935), and the FG-RRT the highest: 0.950 (95% CI 0.948, 0.952). In terms of calibration, Basic-RRT obtained the worst Brier score, 0.0138 (95% CI 0.0130, 0.0146), with the best Brier score for the FG-RRT model, 0.0126 (95% CI 0.0117, 0.0134), although their confidence intervals overlap. Calibration plots for the different approaches for RRT risk estimation are presented in Figures 2, 3 (the latest centered in the 0–5% predicted risk range, which corresponds to 93.7% of the sample according to the FG-RRT model). The overestimation was lower in the FG-RRT model, especially in CKD patients with predicted risk below 5%.

**Table 5:** RRT discrimination and calibration comparison of different KFRE models according to C-statistic and Brier score.

	Discriminative ability: C-statistic 95% conficence interval (95% CI)	Calibration: Brier score 95% conficence interval (95% CI)
Non-USA original KFRE	0.932 (95% CI 0.930, 0.935)	0.0133 (95% CI 0.0125, 0.0141)
Base Hazard adapted-KFRE	0.932 (95% CI 0.930, 0.935)	0.0138 (95% CI 0.0130, 0.0146)
Cox complete reestimation (Cox-RRT)	0.947 (95% CI 0.945, 0.949)	0.0130 (95% CI 0.0122, 0.0138)
Fine and Gray complete reestimation (FG-RRT)	0.950 (95% CI 0.948, 0.952)	0.0126 (95% CI 0.0117, 0.0134)

Discrimination and calibration statistics of the different approaches to estimate risk of KF are presented in Table 6. All models showed good discrimination capacity, with the original KFRE-4 equation presenting the lowest C-statistic, of 0.936 (95% CI 0.934, 0.938), and the FG-RRT presenting the highest, 0.947 (95% CI 0.946, 0.949), just slightly better than the FG-KF. The Basic-RRT model obtained the worst Brier score, 0.0158 (95% CI 0.0149, 0.0168), with the best Brier score for the FG-KF model, 0.0152 (95% CI 0.0143, 0.0161), All Brier Score confidence intervals overlapped.

**Table 6:** KF discrimination and calibration comparison of different KFRE models according to C-statistic and Brier score.

	Discriminative ability: C-statistic 95% conficence interval (95% CI)	Calibration: Brier score 95% conficence interval (95% CI)
Non-USA original KFRE	0.936 (CI 95% 0.934, 0.938)	0.0158 (CI 95% 0.0149, 0.0168)
Base Hazard adapted-KFRE (Basic-RRT)	0.936 (CI 95% 0.934, 0.938)	0.0158 (CI 95% 0.0149, 0.0167)
Cox complete reestimation (Cox-RRT)	0.946 (CI 95% 0.945, 0.948)	0.0153 (CI 95% 0.0144, 0.0162)
Fine and Gray complete reestimation (FG-RRT)	0.947 (CI 95% 0.946, 0.949)	0.0157 (CI 95% 0.0147, 0.0166)
Fine and Gray complete reestimation for KF (FG-KF)	0.946 (CI 95% 0.944, 0.948)	0.0152 (CI 95% 0.0143, 0.0161)

**Figure 4:**
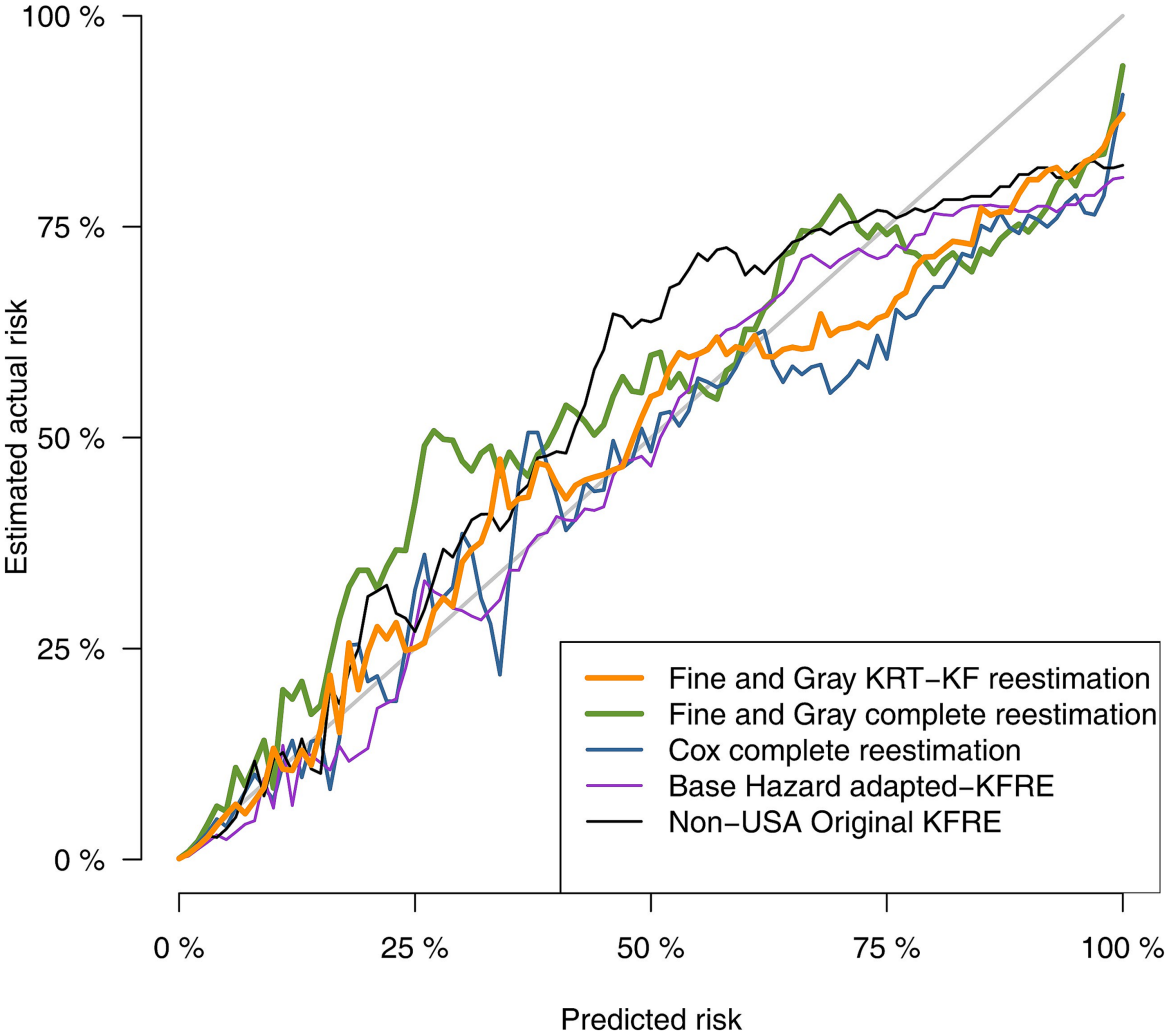
Comparison of predicted vs. observed risk using calibration plot graphical analysis for alternative outcome validation (KF).

**Figure 5:**
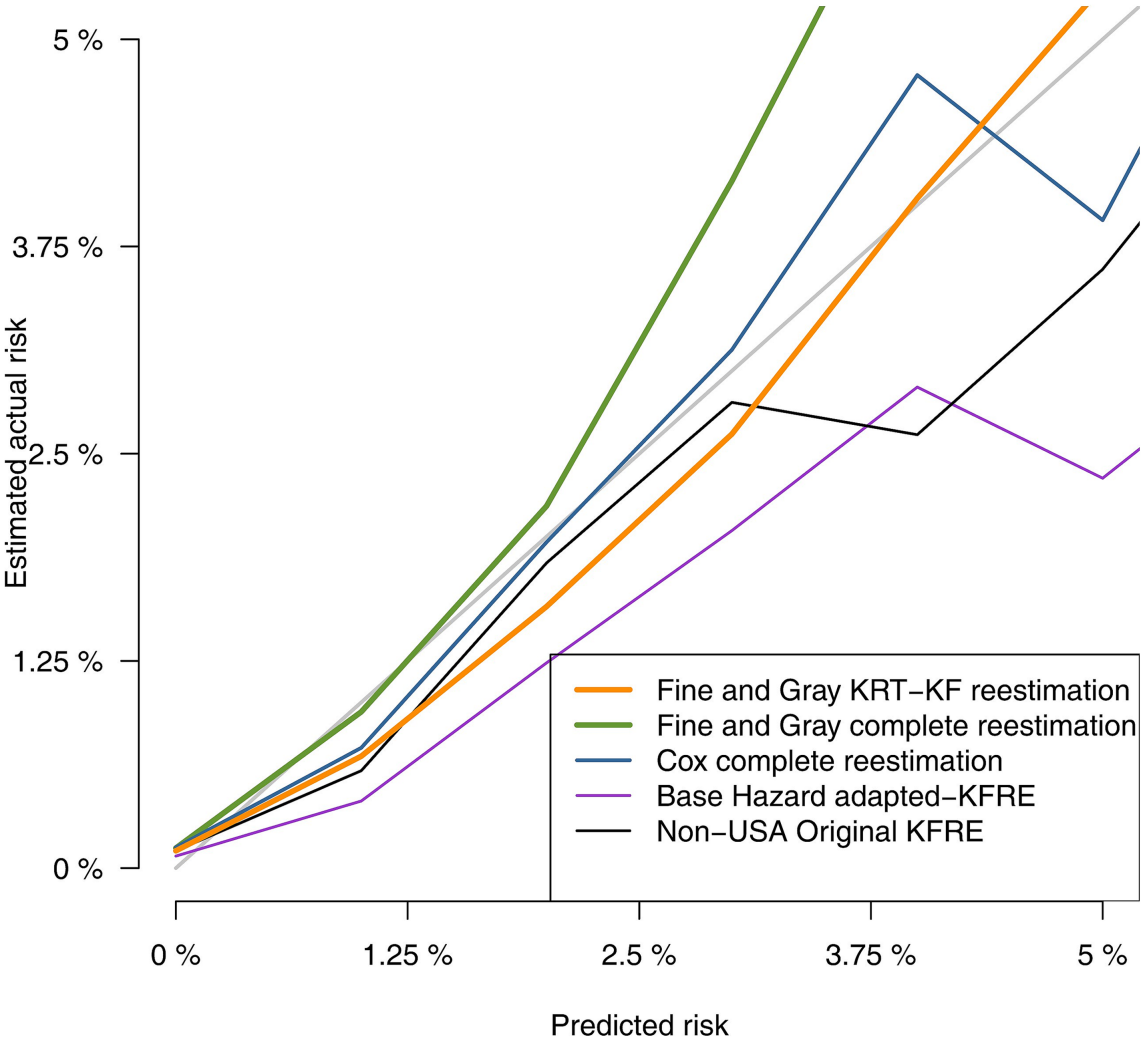
Comparison of predicted vs. observed risk in predicted low risk patients for alternative outcome validation (KF).

Calibration plots for the different approaches for KF risk estimation are presented in Figures 4, 5 (the latest centered in the 0–5% predicted risk range). Figure 5 suggested more stability in both Fine and Gray models in the estimation of predicted risks below 5%. FG-RRT model underpredicted KF risk. FG-KF model corrected underprediction while maintaining stability.

## 4 Discussion

The original KFRE formula for Non-North American cohorts demonstrated excellent discrimination in predicting RRT at 5 years in a European Mediterranean primary care population, but calibration revealed an overestimation of RRT risk, particularly among patients with low predicted risk. Recalibration was necessary, but solely modifying the baseline risk (Basic-RRT) worsened overestimation. Full tailored models - Cox-RRT and FG-RRT - improved KFRE performance by modifying the weight of the risk factors on the equation, age and gender being the most affected variables. FG-RRT model, which accounted for death as a competing risk, yielded the best results. Additionally, KFRE was recalibrated for predicting KF at 5 years, with FG-KF emerging as the most effective model.

### 4.1 The overestimation challenge

The overestimation detected in the original model is consistent with the overestimation reported in previous validation studies of KFRE in population or primary care settings (5, 19, 20, 24).

To validate a risk prediction equation, it is necessary to compare the “observed” actual risk with the model-predicted risk. A key aspect of our study is the estimation of the “observed” outcome during validation. Determining the “observed” actual risk of an adverse event, such as RRT, over a 5-year period involves first analyzing whether we can really observe this outcome occurring or not within that timeframe. When we can clearly determine whether the event has occurred or not without any uncertainty within the specified 5-year period, it is termed an “uncensored binary outcome” (38). However, using this methodology with RRT outcome may lead to errors due to potential loss of follow-up or death during the study. Patients lost to follow-up may or may not receive RRT, and if we categorize them as not receiving it, we may underestimate the true observed risk. Consequently, when comparing it to the predicted risk of the validated tool, we may overestimate the predicted risk. Previous KFRE validation studies are quite inaccurate in this regard but seem to treat the “observed event” as an uncensored binary outcome. This approach might contribute to conclude that KFRE overestimates the RRT risk. In our opinion, estimation of the “observed outcome” for KFRE-4 validation must be performed under the framework of censored observations. The classical Cox method may address this scenario, but it involves treating deceased individuals as censored, which mistakenly assigns them a probability of experiencing RRT risk. Consequently, this approach could inflate the observed outcome by attributing risk probabilities to individuals who are no longer alive (16). As it is presumed that deceased individuals cannot experience the primary outcome, death should also be treated as a competing risk (Fine and Gray) in the estimation of the observed event during the validation process. In our results, despite employing competitive methods for validation, the risk overestimation for the original KFRE persisted. This overestimation was particularly pronounced in patients with low predicted risk. Considering that only 6% of patients exhibited a KFRE risk >5%, recalibrating the formula in our population was crucial for clinical application.

### 4.2 The recalibration process for predicting RRT

We initiated a recalibration process focusing solely on adjusting the baseline hazard of a hypothetical individual (Basic-RRT). This approach mirrored methodologies employed in previous primary care (24, 25) and population-based studies (5, 20). Across these studies, the recalculated hazard for this hypothetical individual consistently showed a decrease compared to the original hazard. Since KFRE was developed using data from a cohort of nephrology-referred patients (4), recalibrating it in cohorts with less severe CKD as it is primary care population, may result in a lower baseline risk for RRT (24). Unexpectedly, our cohort exhibited a recalibrated hazard higher than that observed in non-North American cohorts (5), surpassing even the baseline hazard of the original Canadian model (4). This could be due to the high rate of dialysis and transplantation in Catalonia (39). However, RRT incidence in the present study was not higher than in other primary care KFRE validation studies (23–25). Increasing the baseline risk while assuming that no other variables of the equation were affected resulted in exacerbating the overestimation of RRT risk, indicating an inefficient recalibration.

On the contrary, a comprehensive recalibration model significantly improved the performance of KFRE for determining RRT risk in our population. Cox-RRT and FG-RRT models notably reduced this overprediction. By considering the varying impact of different variables in our population, we achieved better discrimination and calibration. Age and sex played a special role in our population as modifier factors of RRT risk. FG-RRT model revealed that despite an elevated baseline hazard for a hypothetical individual, the different impact of the four variables helps to mitigate it. While a low eGFR or high uACR had a minor effect on modifying RRT risk when comparing it to previous models, being male was associated with a 60% greater impact on RRT risk in our population. However, the most noteworthy aspect of this recalibration is the pivotal role of age in reducing the likelihood of receiving RRT. In our population, this negative influence on RRT risk significantly increased by nearly 300%. In other words, despite the high rate of RRT, older individuals undergo significantly fewer dialysis or transplant procedures in relative terms. This interesting phenomenon may be attributed to the fact that Catalonia, a Mediterranean region with a universal healthcare system, has a higher life expectancy than Canada, where the first model was developed. In 2019 Canadian life expectancy was 82.3 years old for both genders while in Catalonia was 84 (40, 41) years. In a European Mediterranean population with a longer life expectancy and therefore a significant concentration of patients in the more advanced stages of life, resources may be prioritized among younger individuals. Gender inequality may also be slightly higher in our population and women may tend to choose or be offered less RRT (32). Our data show that women received less RRT but do not allow us to know with detail what type of treatment is being chosen or offered: Conservative kidney management (CKM) or no treatment. CKM is the most recommended approach and, in a highly accessible healthcare system as in ours, we believe that most patients may benefit from it. This focuses on improving the quality of life for advanced CKD patients through palliative care, respecting their preferences while forgoing renal replacement therapies but using other medical strategies to delay progression (42–44). These aspects would justify the testing of this newly adapted KFRE version for predicting RRT in some European countries with the same sociological background.

The competing risk approach is relevant in the validation of an instrument but also in the development of a new one. FG-RRT outperformed the Cox model for predicting RRT, showing enhanced discrimination and calibration, particularly in the lower-risk subgroup, diminishing overprediction. This was crucial because most of our population had a KFRE-risk below 5%. Previous literature highlights the tendency of KFRE for risk overestimation, especially in older patients and risks extending beyond 5 years (11, 16, 31). This was attributed to the model’s failure to consider death as a competitive risk (11, 16, 24, 31). Grams addressed this issue in 2023 by developing an equation incorporating both competing risks and eGFR splines and proved better performance, especially in subgroups of elderly and low risk patients (21). Given the elderly population in primary care, applying a competitive risk recalibration was expected to mitigate overestimation. Our findings support previous hypotheses, emphasizing the importance of recalibrating KFRE to include death as a competitive risk in primary care settings.

### 4.3 Recalibrating for predicting KF

As far as we know, for the first time, we developed a new KFRE for predicting KF using competitive risks and compared it to other models in the same primary care cohort. This new tool (FG-KF) exhibited superior performance compared to other equations analyzed in this study at predicting KF, with a good discrimination and calibration ability. Notably, the influence of sex and old age in predicting KF was less pronounced than for predicting RRT. This reflects that women and old people tend to prefer or be offered less RRT as demonstrated in previous publications, being managed in a more conservative manner (32). We detected a 23% higher KF incidence than KRT incidence. Therefore, prioritizing KF as an outcome for KFRE over RRT is warranted to assess CKD progression in primary care.

### 4.4 Relevance to clinical practice

Following our KFRE validation, its application in clinical practice is now feasible for this Mediterranean European primary care population. It could potentially be extended to other Mediterranean populations with similar lifestyles and healthcare systems and long-life expectancy. However, further studies are needed to confirm this. The 2024 KDIGO guidelines recommend healthcare providers to use renal risk assessment to facilitate early identification for disease-modifying therapy, assist in patient education, and set care planning goals (34). A primary care setting is the optimal environment for implementing these recommendations, and KF the most inclusive outcome. KFRE has also been suggested as a tool for guiding referrals from primary care to nephrology specialists (29, 34). Although KFRE has a biological, analytical and instrumentation variability of up to 9% (45), it has been shown in the literature that KFRE risk-based referral can decrease waiting time and improve access to nephrology specialists for high-risk patients without increasing eligibility or healthcare costs (23–28). Positive feedback from both healthcare professionals and patients was received regarding the implementation of KFRE in Canada (46, 47). The thresholds of ≥3% (27, 28), ≥ 5% (24, 26) and ≥ 10% (23, 25) have been shown to be useful as referral criteria in different studies. Therefore, the NICE guidelines 2021 recommend referral to the nephrologist if KFRE ≥5% and/or severe albuminuria (ACR > 619 mg/g) (29), being for the moment the only European guideline that include this criterion (30). We recommend using the formula adapted to the KF outcome, especially in European Mediterranean countries with a long-life expectancy in order not to neglect patients at risk of developing KF without receiving RRT (women and elderly). Even if individuals referred may not undergo RRT, supervision by an experienced nephrologist is also recommended for conservative management.

### 4.5 Strengths and limitations

We consider a strength the large sample size of the study, the strict selection criteria, and the utilization of competing risks to validate different models while using the recommended new CKD-EPI2021 eGFR calculation strategy (26). We validated and recalibrated KFRE-4 for RRT for the first time in a Mediterranean population. We explained the differences and modifications needed to adapt it to sex and life expectancy variations. We also consider it a strength that we recalibrated KFRE-4 for a different outcome (KF) and compared differences between models, justifying the need to prioritize KF as an outcome in primary care at taking clinical decisions.

One methodological limitation is the use of the single-split method, as there are other, more robust, methodologies such as bootstrap cross-validation, albeit less straightforward to explain. Additionally, an important drawback is the potential selection bias in the sample. Only patients with at least one uACR determination were selected, while most of the initial population did not have uACR determined. It is possible that there exists an overrepresentation of diabetic patients, which usually are the patients with most ACR determined.

## 5 Conclusion

KFRE-4 has been validated for predicting RRT and KF in a Mediterranean European Primary care population, demonstrating better performance when recalibrated using competing risk methods. Attending the sex and age peculiarities in this population, predicting for KF offers a more inclusive care, addressing the needs of women and elderly.

## Data Availability

The datasets analysed in this study are not publicly available due to legal reasons related to data privacy protection, but they are available from the corresponding author upon reasonable request. Requests to access the datasets should be directed to bsalvador.apms.ics@gencat.cat.
