# Corrigendum: Recalibrating the kidney failure risk equation for a Mediterranean European population: reducing age and sex inequality

**DOI:** 10.3389/fmed.2025.1583117

**Published:** 2025-03-28

**Authors:** Daniel Bundó-Luque, Oriol Cunillera-Puértolas, Sílvia Cobo-Guerrero, José Romano, Ariadna Arbiol-Roca, José Alberto Domínguez-Alonso, Josep Maria Cruzado, Betlem Salvador-González

**Affiliations:** ^1^CAP Alt Penedès, Gerència d'Atenció Primària i a la Comunitat del Penedès, Institut Català de la Salut, Vilafranca del Penedès, Barcelona, Spain; ^2^Disease, Cardiovascular Risk, and Lifestyle in Primary Health Care (MARCEVAP) Research Group, Fundació Institut Universitari per a la recerca a l'Atenció Primària (IDIAPJGol), L'Hospitalet Llobregat, Barcelona, Spain; ^3^Facultat de Medicina, Universitat de Barcelona (UB), Barcelona, Spain; ^4^Unitat de Suport a la Recerca Metropolitana Sud, Fundació Institut Universitari per a la recerca a l'Atenció Primària de Salut Jordi Gol i Gurina (IDIAPJGol), l'Hospitalet de Llobregat, Barcelona, Spain; ^5^EAP Gavarra, Gerència d'Atenció Primària i a la Comunitat Llobregat, Institut Català de la Salut, Cornellà de Llobregat, Barcelona, Spain; ^6^EAP Sant Josep, Gerència d'Atenció Primària i a la Comunitat Delta Llobregat, Institut Català de la Salut, l'Hospitalet de Llobregat, Barcelona, Spain; ^7^Laboratori Clínic Territorial Metropolitana Sud, Hospital Universitari de Bellvitge, Institut Català de la Salut, l'Hospitalet de Llobregat, Barcelona, Spain; ^8^CAP Vilafranca Nord, Gerència d'Atenció Primària i a la Comunitat del Penedès, Institut Català de la Salut, Vilafranca del Penedès, Barcelona, Spain; ^9^Department of Nephrology, Hospital Universitari Bellvitge, l'Hospitalet de Llobregat, Barcelona, Spain; ^10^Nephrology and Renal Transplantation Group, Bellvitge Institute for Biomedical Research (IDIBELL), l'Hospitalet de Llobregat, Barcelona, Spain

**Keywords:** kidney failure, KFRE, risk assesement, age, sex, competing risk

In the published article, there was an error in affiliation 10. Instead of “Department of Clinical Sciences, University of Barcelona, l'Hospitalet de Llobregat, Barcelona, Spain.” it should be “Facultat de Medicina, Universitat de Barcelona (UB), Barcelona, Spain”

In the published article, there was an error regarding the affiliations for Daniel Bundó-Luque. As well as having affiliation(s) 1 and 2, another affiliation is required: “Facultat de Medicina, Universitat de Barcelona (UB), Barcelona, Spain”.

The corrected version of the affiliations section is:

Daniel Bundó-Luque^1, 2, 3†^, Oriol Cunillera-Puértolas^2, 4†^, Sílvia Cobo-Guerrero^2, 5^, José Romano^2, 6^, Ariadna Arbiol-Roca^2, 7^, José Alberto Domínguez-Alonso^2, 8^, Josep Maria Cruzado^3, 9, 10^ and Betlem Salvador-González^2, 4, *^

^1^CAP Alt Penedès, Gerència d'Atenció Primària i a la Comunitat del Penedès, Institut Català de la Salut, Vilafranca del Penedès, Barcelona, Spain

^2^Disease, Cardiovascular Risk, and Lifestyle in Primary Health Care (MARCEVAP) Research Group, Fundació Institut Universitari per a la recerca a l'Atenció Primària (IDIAPJGol), L'Hospitalet Llobregat, Barcelona, Spain

^3^Facultat de Medicina, Universitat de Barcelona (UB), Barcelona, Spain

^4^Unitat de Suport a la Recerca Metropolitana Sud, Fundació Institut Universitari per a la recerca a l'Atenció Primària de Salut Jordi Gol i Gurina (IDIAPJGol), l'Hospitalet de Llobregat, Barcelona, Spain

^5^EAP Gavarra, Gerència d'Atenció Primària i a la Comunitat Llobregat, Institut Català de la Salut, Cornellà de Llobregat, Barcelona, Spain

^6^EAP Sant Josep, Gerència d'Atenció Primària i a la Comunitat Delta Llobregat, Institut Català de la Salut, l'Hospitalet de Llobregat, Barcelona, Spain

^7^Laboratori Clínic Territorial Metropolitana Sud, Hospital Universitari de Bellvitge, Institut Català de la Salut, l'Hospitalet de Llobregat, Barcelona, Spain

^8^CAP Vilafranca Nord, Gerència d'Atenció Primària i a la Comunitat del Penedès, Institut Català de la Salut, Vilafranca del Penedès, Barcelona, Spain

^9^Department of Nephrology, Hospital Universitari Bellvitge, l'Hospitalet de Llobregat, Barcelona, Spain

^10^Nephrology and Renal Transplantation Group, Bellvitge Institute for Biomedical Research (IDIBELL), l'Hospitalet de Llobregat, Barcelona, Spain

In the published article, there was a typographical error in the definition of the KFRE formula, although the formula has been correctly interpreted and used throughout the article.

A correction has been made to “Results” section, “3.3 New KFRE development” sub-section, first paragraph. This sentence previously stated:

“All adjusted models provide an estimation of the 5-year risk according to the following general formula:


$$Risk=base.hazard^{\exp}\left(\begin{array}{l} coef_{\left(age/10\right)}\times \left(\frac{age}{10}-7.036\right)+\\ coef_{\left(sex=male\right)}\times \left(I-0.5642\right)+\\ coef_{\left(eGFR/5\right)}\times \left(\frac{eGFR}{5}-7.222\right)+\\ coef_{\left(\ln\left(uACR\right)\right)}\times \left(\ln\left(uACR\right)-5.137\right) \end{array}\right)$$

*I*= *1 if male and 0 if female*

The base hazard and coefficients (marginal effects) were estimated for the different models”.

The corrected sentence appears below:

“All adjusted models provide an estimation of the 5-year risk according to the following general formula:


$$Risk=1-base.survival^{\exp}\left(\begin{array}{l} coef_{\left(age/10\right)}\times \left(\frac{age}{10}-7.036\right)+\\ coef_{\left(sex=male\right)}\times \left(I-0.5642\right)+\\ coef_{\left(eGFR/5\right)}\times \left(\frac{eGFR}{5}-7.222\right)+\\ coef_{\left(\ln\left(uACR\right)\right)}\times \left(\ln\left(uACR\right)-5.137\right) \end{array}\right)$$

*I*= *1 if male and 0 if female*

*Base.hazard*= *1 – base.survival*

The base hazard and coefficients (marginal effects) were estimated for the different models”.

The authors apologize for this error and state that this does not change the scientific conclusions of the article in any way. The original article has been updated.

